# Proteomic Characterization and Comparison of Malaysian *Tropidolaemus wagleri* and *Cryptelytrops purpureomaculatus* Venom Using Shotgun-Proteomics

**DOI:** 10.3390/toxins8100299

**Published:** 2016-10-18

**Authors:** Syafiq Asnawi Zainal Abidin, Pathmanathan Rajadurai, Md Ezharul Hoque Chowdhury, Muhamad Rusdi Ahmad Rusmili, Iekhsan Othman, Rakesh Naidu

**Affiliations:** 1 Jeffrey Cheah School of Medicine and Health Sciences, Monash University Malaysia, Jalan Lagoon Selatan, Bandar Sunway, Selangor Darul Ehsan 47500, Malaysia; sazai1@student.monash.edu (S.A.Z.A.); drpathma@gmail.com (P.R.); md.ezharul.hoque@monash.edu (M.E.H.C.); kdrakeshna@hotmail.com (R.N.); 2 Ramsay Sime Darby Healthcare, Sime Darby Medical Centre, No. 1, Jalan SS12/1A, Subang Jaya, Selangor Darul Ehsan 47500, Malaysia; 3 Kuliyyah of Pharmacy, International Islamic University Malaysia, Kuantan Campus, Bandar Indera Mahkota, Kuantan, Pahang Darul Makmur 25200, Malaysia; rusdirusmili@iium.edu.my

**Keywords:** *Tropidolaemus wagleri*, *Cryptelytrops purpureomaculatus*, proteome, tandem mass spectrometry

## Abstract

*Tropidolaemus wagleri* and *Cryptelytrops purpureomaculatus* are venomous pit viper species commonly found in Malaysia. Tandem mass spectrometry analysis of the crude venoms has detected different proteins in *T. wagleri* and *C. purpureomaculatus*. They were classified into 13 venom protein families consisting of enzymatic and nonenzymatic proteins. Enzymatic families detected in *T. wagleri* and *C. purpureomaculatus* venom were snake venom metalloproteinase, phospholipase A_2_, l-amino acid oxidase, serine proteases, 5′-nucleotidase, phosphodiesterase, and phospholipase B. In addition, glutaminyl cyclotransferase was detected in *C. purpureomaculatus*. C-type lectin-like proteins were common nonenzymatic components in both species. Waglerin was present and unique to *T. wagleri*—it was not in *C. purpureomaculatus* venom. In contrast, cysteine-rich secretory protein, bradykinin-potentiating peptide, and C-type natriuretic peptide were present in *C. purpureomaculatus* venom. Composition of the venom proteome of *T. wagleri* and *C. purpureomaculatus* provides useful information to guide production of effective antivenom and identification of proteins with potential therapeutic applications.

## 1. Introduction

Snake venom is a highly complex mixture of proteins and polypeptides with a myriad of biological activities. It functions as an important tool for defense against predators, prey immobilization, and facilitation of prey digestion [1]. There are two families of terrestrial venomous snake that cause the majority of envenoming cases in Malaysia: Elapidae and Viperidae. Among the potentially dangerous snakes that belong to the viperid subfamily Crotalinae (pit vipers) are *Tropidolaemus wagleri* (Temple or Wagler’s pit viper) and *Cryptelytrops purpureomaculatus* (mangrove pit viper) [1]. Envenomation from these pit vipers is often associated with intense pain, local swelling, necrosis, hemorrhage, and blood pressure disruption [1].

*Tropidolaemus wagleri* is recognized as a primitive and unique pit viper, morphologically as well as biochemically [2,3]. Enzymatic properties of the venom highlight similarity of venom components to other vipers and include nonlethal enzymes such as phosphodiesterase, thrombin-like enzyme (TLE), l-amino acid oxidase (LAAO), and phospholipase A_2_ (PLA_2_) [4]. Lethal components of *T. wagleri* venom were identified as low molecular-weight toxins, namely waglerins-1, -2, -3, and -4 [5]. These toxins were classified as neurotoxins due to their competitive antagonism with the nicotinic acetylcholine receptor (nAChR) [6,7]. This feature is considered unusual for snakes of the family Viperidae, as the presence of postsynaptic neurotoxins is well-known in the venoms of elapid snakes [8]. *Cryptelytrops purpureomaculatus* is known to be very aggressive and is commonly found on mangrove mudflats on the coastal region of west peninsular Malaysia. The color of *C. purpureomaculatus* is variable, ranging from grayish to olive and brownish purple [1,9]. The venom is known to be nonfatal to humans but causes local swelling and pain [10,11]. The venom was found to have anticoagulant, thrombin-like, hemorrhagic, and other enzymatic activities, and it is more toxic than several other Asian arboreal viper species [12].

Information on the venom proteome is important for understanding and predicting the clinical consequences of envenomation and for formulating an effective antivenom that will target and neutralize venom components common to viper species [13]. At present, online protein database searches using UniProt [14] for “*Tropidolaemus wagleri*” and “*Cryptelytrops purpureomaculatus*” listed only five and four proteins, respectively. Hence, much is still unknown about the proteome of *T. wagleri* and *C. purpureomaculatus* venoms. Here we performed proteomic profiling and compared the crude venom composition of *T. wagleri* and *C. purpureomaculatus* using a shotgun-proteomics approach. Shotgun-proteomics and liquid chromatography tandem mass spectrometry (LC–MS/MS) have been described as a rapid, bottom-up proteomic techniques that allows direct analysis of simple and complex protein samples to generate a global profile of the total protein components [15,16].

## 2. Results and Discussion

### 2.1. The Venom Proteome of T. wagleri and C. purpureomaculatus

The venom proteomes of *T. wagleri* and *C. purpureomaculatus* have never been completely reported using proteomic techniques such as shotgun-proteomic and LC–MS/MS. Shotgun-proteomics and LC–MS/MS approaches have been used to characterize many other snake venom proteomes, including kraits and cobras [17,18].

#### 2.1.1. *T. wagleri* Venom

Earlier studies on *T. wagleri* venom have demonstrated the presence of enzymatic proteins commonly occurring in pit viper venom such as phosphodiesterase, phosphomonoesterase, hydrolase, TLE, LAAO, and PLA_2_ [4]. The venom lethality was comparable to other pit viper species venom but distinct due to the lack of hemorrhagic activity [4,5]. The lethal toxins were then identified as low molecular-weight peptides; waglerins [4,5]. In the public protein database (UniProtKB), only five proteins were reviewed and listed under the heading of *Tropidolaemus wagleri*: waglerin-3 (WAG13_TROWA), SNACLEC trowaglerix subunit alpha (SLA_TROWA), waglerin-4 (WAG24_TROWA), SNACLEC trowaglerix subunit beta (SLB_TROWA), and NADH-ubiquinone oxidoreductase (NU4M_TROWA). In our study, mass spectrometry analysis of *T. wagleri* venom detected 13 different proteins that shared similar sequences with proteins in the database (SwissProt, Serpentes) (Table 1). Protein families that were not reported previously in the venom, (e.g., snake venom metalloproteinase (SVMP)) were detected in the present study (Table 2).

#### 2.1.2. *C. purpureomaculatus* Venom

The enzymatic activities detected in *C. purpureomaculatus* venom are due to the presence of TLE, phospholipase A, arginine ester hydrolase, arginine amidase, protease, 5′-nucleotidase, acetylcholinesterase, and alkaline phosphomonoesterase [19]. The venom may exhibit its lethality through hemorrhagic, edema-inducing, and thrombin-like activity [19]. There were only four proteins that were deposited under *Cryptelytrops purpureomaculatus* in UniProt, namely, TLE purpurase (VSPP_TRIPP), SNACLEC purpureotin subunit alpha (SLA_TRIPP) and beta (SLB_TRIPP), and CRVP (CRVP_TRIPP). In this study, 51 different venom proteins with sequence similarity with proteins in the public protein database were detected (Table 1). Several proteins that have never been reported previously for *C. purpureomaculatus* such as SVMP-disintegrin, glutaminyl-peptide cyclotransferase (QPCT), bradykinin-potentiating and C-type natriuretic peptide (BCNP), and phospholipase B (PLB) were detected in this study (Table 2).

### 2.2. Venom Composition of T. wagleri and C. purpureomaculatus

**Figure 1:**
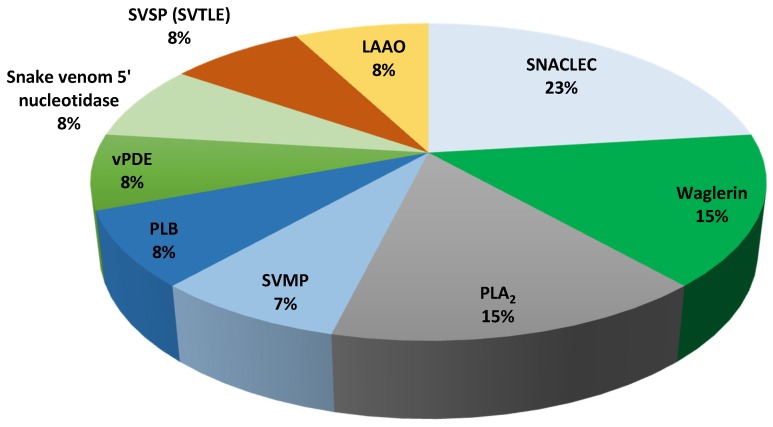
Relative abundance of venom proteins that were detected using LC–MS/MS from Malaysian *T. wagleri* venom. PLB: phospholipase B, vPDE: venom phosphodiesterase, SVTLE: snake venom thrombin-like enzyme, SVSP: snake venom serine protease, SNACLEC: snake venom C-type lectin, LAAO: l-amino acid oxidase, PLA_2_: phospholipase A_2_, SVMP: snake venom metalloproteinase.

**Figure 2:**
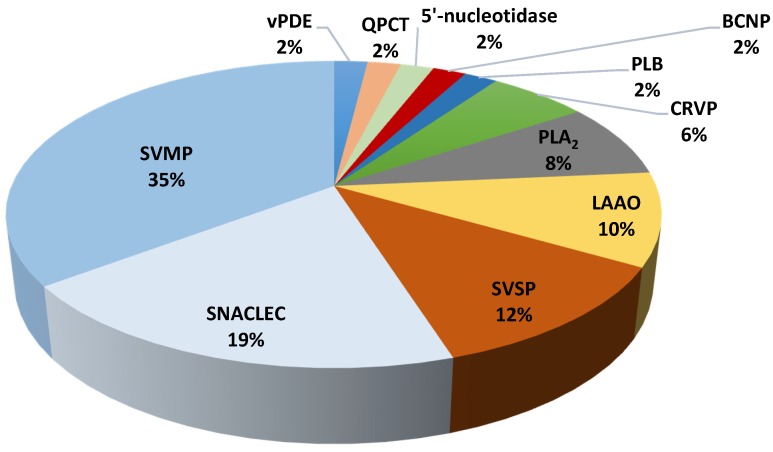
Relative abundance of venom proteins that were detected using LC–MS/MS from Malaysian *C. purpureomaculatus* venom. PLB: phospholipase B, vPDE: venom phosphodiesterase, SVSP: snake venom serine protease, QPCT: glutaminyl-peptide cyclotransferase, BCNP: bradykinin-potentiating and C-type natriuretic peptide, CRVP: cysteine-rich venom protein, PLA_2_: phospholipase A_2_, LAAO: l-amino acid oxidase, SNACLEC: snake venom C-type lectin, SVMP: snake venom metalloproteinase.

The venom composition of *T. wagleri* and *C. purpureomaculatus* was divided into enzymatic and nonenzymatic protein families (Table 3 and Table 4). Enzymatic protein families were found to be the major venom protein family in *T. wagleri* and *C. purpureomaculatus* venom with 62% and 73% of the total proteins, respectively (Figure 1 and Figure 2). Nonenzymatic protein families of *T. wagleri* and *C. purpureomaculatus* venom were detected at 38% and 27% of the total proteins, respectively.

#### 2.2.1. Enzymatic Protein Families of *T. wagleri* and *C. purpureomaculatus* Venom

SVMP was identified to be 7% of the total proteins detected in *T. wagleri* (Table 5). In contrast, it is the major venom protein family representing 35% of the total proteins in *C. purpureomaculatus*, 5 times more compared to *T. wagleri* (Table 5). The high abundance of SVMP in *C. purpureomaculatus* is consistent with the biochemical studies on the venom [19,20]. SVMPs are one of the most important components of the viperid venoms, causing hemorrhage by affecting blood coagulation and/or integrity of extracellular matrix components such as collagen, laminin, and fibronectin [21,22,23]. SVMPs are classified into three classes, P-I to P-III, based on their domain structure [24]. The majority of SVMPs that were detected in both *T. wagleri* and *C. purpureomaculatus* belong to P-II and P-III classes (Table 1 and Table 2). P-II class contains metalloproteinase and disintegrin domains while P-III class contains metalloproteinase, disintegrin-like, and cysteine-rich domains [24]. Biological activities of P-II SVMPs include proteolytic induction [25], platelet aggregation inhibition [26], and hemorrhagic [27]. Most P-III SVMPs were identified as hemorrhagic and have the most potent biological activity among the three SVMP classes [24]. Additionally, some P-III SVMPs can induce apoptosis in vascular endothelial cells [28,29,30]. The detection of SVMP in *T. wagleri* is intriguing, as several earlier reports have shown the absence of hemorrhage activity from the venom [4,31]. Higher sensitivity and resolution of LC–MS/MS compared to biochemical methods could be the reason for the detection of SVMPs in the *T. wagleri* venom. SVMPs from *T. wagleri* have never been isolated and studied, therefore there is a need to study these SVMPs and elucidate their biological activity and function. It is possible that intraspecific snake venom variations and regional variations could contribute toward the presence of SVMPs in the venom [32,33]. More extensive study using samples from various regions where *T. wagleri* is endemic is required to confirm this possibility.

LAAO and PLA_2_ were other major components in the venoms (Table 5). LAAO and PLA_2_ activities of *T*. *wagleri* and *C. purpureomaculatus* venoms have been described in other studies [4,19]. LAAO is a ubiquitous component of snake venom, but its abundance varies between species [34]. This was demonstrated by our present findings where the percentage of LAAO was found to be lower in *T. wagleri* (8%), compared to *C. purpureomaculatus* (10%) (Table 5). The LAAOs that were detected in this study showed sequence similarity with LAAO from several genera including *Calloselasma*, *Gloydius*, *Viridovipera*, *Bothrops*, *Daboia*, and *Cerastes* (Table 1 and Table 2). LAAOs in *T. wagleri* and *C. purpureomaculatus* could be partially or synergistically responsible for various biological effects upon envenomation, including hemorrhage [35], edema [36], and platelet aggregation [37,38]. Venom PLA_2_ may interrupt normal physiological processes, causing various pharmacological effects such as neurotoxicity, myotoxicity, and cardiotoxicity [39,40,41]. The PLA_2_ isoforms that were detected in *T. wagleri* and *C. purpureomaculatus* venom showed sequence similarity with PLA_2_ that were described in the other viper species venoms including *Bothrops*, *Trimeresurus*, and *Ovophis* venoms (Table 1 and Table 2). PLA_2_ detected from both species were identified as basic and acidic isoforms of PLA_2_ (Table 1 and Table 2). The presence of PLA_2_ isoforms in *T. wagleri* was consistent with the discovery of acidic and basic variants from Sulawesi and Sumatran origin *Tropidolaemus* genus [42].

Other families of enzymatic proteins detected in *T. wagleri* and *C. purpureomaculatus* include snake venom 5′-nucleotidase, venom phosphodiesterase (vPDE), and PLB. Each of these proteins was detected at 8% in *T. wagleri* and 2% in *C. purpureomaculatus* (Table 5). Snake venom serine protease (SVSP) was determined at 8% in *T. wagleri* and 12% in *C. purpureomaculatus* (Table 5). Glutaminyl-peptide cyclotransferase (QPCT) was demonstrated in *C. purpureomaculatus* at 2%, but was not found in *T. wagleri* (Table 5). Snake venom QPCT has been suggested to have an indirect contribution to venom toxicity via posttranslational modification of venom proteins [43,44]. QPCT is important in the *N*-terminal glutamine cyclization that induces toxin maturation, protects from exopeptidase degradation, and/or assists proper protein conformation [44].

The presence and activity of snake venom 5′-nucleotidase and vPDE in *T. wagleri* and *C. purpureomaculatus* have been demonstrated by biochemical assays, which agrees with our findings [4,19]. LC–MS/MS data showed that these proteins shared sequence similarity to proteins found in *Gloydius brevicaudus* and *Crotalus adamanteus* (Table 1 and Table 2). These proteins were thought to inhibit platelet aggregation through the liberation of purines using endogenous source from their envenomed victims [45]. We have also detected the presence of PLB in *T. wagleri* and *C. purpureomaculatus* crude venoms, similar to those found in *Crotalus adamanteus* (Table 1 and Table 2). The role of PLB in snake venom was not well understood, but its hemolytic activity has been demonstrated in vitro [46]. SVSP has several biological activities such as platelet aggregation, coagulation, and fibrinolysis [47,48]. In this study, different classes of SVSP were detected from *T. wagleri* and *C. purpureomaculatus* including TLE, fibrinogenase, and vPA (Table 1 and Table 2). TLE plays a significant role in coagulation process [49]. TLE has been purified and characterized from *C. purpureomaculatus*, but not from *T. wagleri* [50]. The antibody developed from purpurase, a TLE isolated from *C. purpureomaculatus*, was found to strongly react with *Trimeresurus* complex venom, suggesting sequence homology of TLEs within the complex [50]. This finding is consistent with our LC–MS/MS data that detected TLEs that shared similar sequences with TLEs found in *Cryptelytrops albolabris*, and *Viridovipera stejnegeri* (Table 2). Fibrinogenase affects the blood clotting mechanism through fibrinolytic action [51,52]. vPA is another class of serine protease that activates plasminogen to plasmin, promoting fibrinolysis upon envenomation of its victim [53].

#### 2.2.2. Nonenzymatic Protein Families of *T. wagleri* and *C. purpureomaculatus*

Snake venom C-type lectin (SNACLEC) was found to be the largest nonenzymatic protein family and accounted for 23% and 19% of *T. wagleri* and *C. purpureomaculatus* crude venom, respectively (Table 5). The SNACLECs in *T. wagleri* venom shared similarity with SNACLEC found in *Ophiophagus hannah*, *Viridovipera stejnegeri*, and *Cryptelytrops albolabris* venoms (Table 1). The SNACLECs found in *C. purpureomaculatus* shared sequence similarity with SNACLEC found in genus *Trimeresurus*, *Deinagkistrodon acutus*, and *Echis multisquamatus* (Table 2). SNACLEC from both *T. wagleri* (trowaglerix) and *C. purpureomaculatus* (purpureotin) have been characterized previously [54,55]. These proteins were known to either inhibit or activate specific platelet receptors, such as integrins, which affect thrombosis or hemostasis processes [56,57]. Trowaglerix induces platelet aggregation through specific binding to glycoprotein IV (GPIV) receptor [54], while purpureotin binds to glycoprotein Ib (GPIb) receptor [57].

Waglerin was found exclusively in *T. wagleri* and it is the second largest protein family, which constitutes 15% of the crude venom (Figure 1, Table 5). Waglerin has been well characterized and considered as *T. wagleri*’s most unique and most lethal protein [5,7,58,59]. To date, four types of waglerins have been described [5,58]. The peptides waglerins-1 and -2 differ by one amino acid at position 10 (waglerin-1: histidine, waglerin-2; tyrosine). Waglerins-3 and -4 are almost homologous to waglerins-1 and -2, respectively, except for two additional amino acids (serine and leucine) at their *N*-terminal [5,58]. Waglerins identified in this study were noted as waglerins-3 and -4 because the database used in this study indicated that waglerins-1 and -2 corresponded with waglerins-3 and -4, respectively (Table 1). Waglerin exerts its toxicity through competitive antagonism with the nicotinic acetylcholine receptor (nAChR) at nanomolar concentrations [7,58]. Earlier studies found that waglerin-1 selectivity binds to the α-ε nAChR interface with 2000-fold higher affinity compared to other binding sites [6]. The finding of high abundance of waglerin in our study (15%) agrees with several reports that identified the protein as the major toxic component of the venom [4,5,7]. In addition, the percentage of peptide coverage identified by MS spectra correlates with the protein abundance [60]. This data is supported by LC–MS/MS, which showed 100% waglerin peptide detection coverage (Table 1, Supplementary File 1).

Cysteine-rich venom protein (CRVP), also known as snake venom cysteine-rich secretory protein (CRISP) and BCNP were found specifically in *C. purpureomaculatus* at 2% of the total venom (Table 5). The amino acid sequence of CRVP from various snake species has been well characterized, however, the biological activity has not been fully understood [61]. Nonetheless, CRVP isolated from several viper species may exhibit neurotoxicity through the blocking of several ion channels [62,63]. Three different CRVPs were detected from *C. purpureomaculatus* venom using LC–MS/MS. These CRVPs shared sequence similarity with CRVP from *Viridovipera stejnegeri*, *Protobothrops flavoviridis* and a CRVP from *C. purpureomaculatus*, tripurin (Table 2). BCNP is a unique snake venom protein, and its cDNA encodes both bradykinin-potentiating peptide (BPP) and C-type natriuretic peptides (CNP), thereby integrating two different vasodilating molecules into one [64]. Biological effects of BCNP upon envenomation include hypotension and loss of consciousness [65,66]. In this study, BCNP was found specifically in *C. purpureomaculatus* at 2% of the total venom (Table 5). BPPs can inhibit angiotensin-converting enzyme activity and, along with CNP, have been investigated for the treatment of human hypertension and several other cardiovascular diseases, including congestive heart failure [65,66,67]. We believe this is the first study to identify the presence of BCNP in *C. purpureomaculatus* venom.

## 3. Conclusions

We have successfully characterized and compared the venom proteome of Malaysian *T. wagleri* and *C. purpureomaculatus* by using shotgun-proteomics, LC–MS/MS, and protein de novo sequencing. The present data have revealed the complex composition of the crude venom from both species. The venom proteome of both snakes consists of enzymatic and nonenzymatic protein families. The proteins detected in both *T. wagleri* and *C. purpureomaculatus* were SVMP, SVSP, LAAO, PLA_2_, vPDE, snake venom 5′-nucleotidase, and SNACLEC. Neurotoxin waglerin was unique in *T. wagleri* venom, whereas BCNP and QPCT were unique in *C. purpureomaculatus* venom. This information may be useful to predict the clinical prognosis after envenoming and provide better guidance for the production of effective antivenom. Moreover, proteins detected in *T. wagleri* and *C. purpureomaculatus* venoms could be selectively investigated for therapeutic potentials.

## 4. Materials and Methods

### 4.1. Materials

#### Snake Venom

Crude *T. wagleri* and *C. purpureomaculatus* venoms were obtained from Mr. Zainuddin Ismail, a private snake enthusiast from Perlis in Peninsula Malaysia. All snakes originated from the west of Peninsular Malaysia. Snake venoms were collected in a sterile container covered with parafilm. The venoms were transported back to Monash Malaysia campus on ice and frozen at −80 °C before subjecting them to the freeze-drying process. Freeze dried venom was weighed, labeled, and stored at −20 °C until use. Venom samples from three different sampling sessions were used for the experiment.

### 4.2. Methods

#### 4.2.1. In-Solution Tryptic Digestion

Approximately 0.5 mg of crude venom was added into 1.5 mL tube in triplicates and mixed with 25 μL of 100 mM ammonium bicarbonate (ABC), 25 μL of trifluoroethanol, and 1 μL of 200 mM 1,4-dithiothreitol (DTT). The mixture was then briefly vortexed, and incubated at 60 °C for 1 h. Next, the protein in the tube was alkylated by adding 4.0 μL of 200 mM iodoacetamide, briefly vortexed, and incubated at room temperature in the dark (covered with aluminum foil) for 1 h. Subsequently, 1 μL of 200 mM DTT was added to the tube and incubated at room temperature in the dark for another 1 h. Double-distilled water and 100 mM ABC were then added to the sample mixture to dilute the protein denaturant and raised the pH to 7–9. Trypsin solution was added to the tubes at the weight ratio of 1:50, briefly vortexed and incubated overnight at 37 °C. On the next day, 1 μL of formic acid was added to stop the trypsin digestion, briefly vortexed, and left in a vacuum concentrator overnight to concentrate the digested proteins. Samples were kept at −20 °C prior to LC–MS/MS analysis.

#### 4.2.2. Nanoflow Liquid Chromatography Electrospray-Ionization Coupled with Tandem Mass Spectrometry (Nanoflow–ESI–LC–MS/MS)

The digested peptides were loaded into an Agilent C18 300 Å Large Capacity Chip (Agilent, Santa Clara, CA, USA) column that was equilibrated with 0.1% formic acid in water (solution A). The peptides were eluted from the column with 90% acetonitrile in 0.1% formic acid in water (solution B) using the following gradient; 3%–50% solution B over 0–30 min, 50%–95% solution B over 2 min, 95% solution B for 7 min, and 95%–3% solution B over 39–47 min. Quadrupole-time of flight (Q-TOF) polarity was set at positive with capillary and fragmenter voltage being set at 2050 V and 300 V, respectively, and 5 L/min of gas flow with a temperature of 300 °C. The peptide spectrum was analyzed in auto MS mode ranging from 110–3000 *m*/*z* for MS scan and 50–3000 *m*/*z* for MS/MS scan. The spectrum was then analyzed with Agilent MassHunter (Agilent Technologies, Santa Clara, CA, USA) data acquisition software and then PEAKS 7.0 software (Bioinformatics Solutions Inc., Waterloo, ON, Canada).

#### 4.2.3. Venom Protein Identification by Automated de Novo Sequencing (PEAKS Studio 7.0)

Protein identification by automated de novo sequencing was performed with PEAKS Studio 7.0 (Bioinformatics Solution Inc., Waterloo, ON, Canada). SwissProt.Serpentes (May 2015) database was used for protein identification and homology search by comparing the de novo sequence tag. Carbamidomethylation was set as fixed modification with maximum mixed cleavages at 3. Parent mass and fragment mass error tolerance were both set 0.1 Da with monoisotopic as the precursor mass search type. Trypsin was selected as the enzyme used for digestion. False discovery rate (FDR) of 1% and unique peptide ≥2 were used for filtering out inaccurate proteins. A −10lgP score of greater than 20 indicates detected proteins are relatively high in confidence as it targets very few decoy matches above that threshold [68]. The percentage of the venom protein family in the crude venom was calculated using the following formula:

no.of proteins (protein family)total proteins detected using LC−MS/MS ×100

## Figures and Tables

**Table 1:** List of detected proteins in Malaysian *T. wagleri* from in-solution digests by LC–MS/MS. Please refer to Supplementary File 1 for a complete list of peptides and *m*/*z* values.

Accession	−10lgP	Coverage (%)	#Peptides	#Unique	Description
**P58930|WAG24_TROWA**	318.99	100	23	21	Waglerin-4 (*Tropidolaemus wagleri*)
**P24335|WAG13_TROWA**	306	100	20	18	Waglerin-3 (*Tropidolaemus wagleri*)
**C5H5D3|VM32_BOTAT**	247.11	16	6	2	Zinc metalloproteinase-disintegrin-like batroxstatin-2 (*Bothrops atrox*)
**P81382|OXLA_CALRH**	220.52	15	7	3	l-amino-acid oxidase (*Calloselasma rhodostoma*)
**F8S101|PLB_CROAD**	164.24	8	4	3	Phospholipase B (*Crotalus adamanteus*)
**P0C8J0|SLA_OPHHA**	161.12	85	4	4	Snaclec ophioluxin subunit alpha (Fragment) (*Ophiophagus hannah*)
**Q71RR4|SLA_TRIST**	78.55	17	2	2	Snaclec coagulation factor IX/factor X-binding protein subunit A (*Viridovipera stejnegeri*)
**P0DM38|SLDA_TRIAB**	65.57	20	2	2	Snaclec alboaggregin-D subunit alpha (*Cryptelytrops albolabris*)
**J3SBP3|PDE2_CROAD**	125.42	15	7	7	Venom phosphodiesterase 2 (*Crotalus adamanteus*)
**B6EWW8|V5NTD_GLOBR**	118.86	14	6	6	Snake venom 5′-nucleotidase (*Gloydius brevicaudus*)
**P00625|PA2A1_OVOOK**	111.35	16	4	3	Acidic phospholipase A_2_ DE-I (*Ovophis okinavensis*)
**P0DM51|PA2B1_BOTPA**	67.6	24	3	3	Basic phospholipase A_2_ BnpTX-1 (Fragment) (*Bothrops pauloensis*)
**O13069|VSP2_BOTJA**	92.72	21	4	3	Thrombin-like enzyme KN-BJ 2 (*Bothropoides jararaca*)

**Table 2:** List of detected proteins in Malaysian *C. purpureomaculatus* from in-solution digests by LC–MS/MS. Please refer to Supplementary File 2 for a complete list of peptides and *m*/*z* values.

Accession	−10lgp	Coverage (%)	#Peptides	#Unique	Description
**P0C6E8|VM3G1_TRIGA**	322.01	38	29	5	Zinc metalloproteinase/disintegrin (Fragment) (*Trimeresurus gramineus*)
**P15503|VM2TA_TRIGA**	306.48	33	24	2	Zinc metalloproteinase/disintegrin (*Trimeresurus gramineus*)
**Q3HTN1|VM3SA_TRIST**	276.23	30	15	8	Zinc metalloproteinase-disintegrin-like stejnihagin-A (*Viridovipera stejnegeri*)
**P62383|VM2G_TRIGA**	270.15	85	6	2	Disintegrin trigramin-gamma (*Trimeresurus gramineus*)
**Q2LD49|VM3TM_TRIST**	255.16	23	15	6	Zinc metalloproteinase-disintegrin-like TSV-DM (*Viridovipera stejnegeri*)
**P0C6B6|VM2AL_TRIAB**	246.22	24	14	8	Zinc metalloproteinase homolog-disintegrin albolatin (*Cryptelytrops albolabris*)
**P0DM87|VM2_TRIST**	242.76	11	10	3	Zinc metalloproteinase-disintegrin stejnitin (*Viridovipera stejnegeri*)
**Q3HTN2|VM3SB_TRIST**	235.08	13	7	3	Zinc metalloproteinase-disintegrin-like stejnihagin-B (*Viridovipera stejnegeri*)
**Q8AWX7|VM2AG_GLOHA**	224.09	12	6	2	Zinc metalloproteinase-disintegrin agkistin (*Gloydius halys*)
**P20164|VM3HB_PROFL**	214.8	14	10	5	Zinc metalloproteinase-disintegrin-like HR1b (*Probothrops flavoviridis*)
**Q2UXQ5|VM3E2_ECHOC**	202.85	6	4	2	Zinc metalloproteinase-disintegrin-like EoVMP2 (*Echis ocellatus*)
**Q98UF9|VM3H3_BOTJA**	180.86	12	5	2	Zinc metalloproteinase-disintegrin-like HF3 (*Bothropoides jararaca*)
**Q0NZX6|VM2JR_BOTJA**	175.59	13	4	4	Zinc metalloproteinase-disintegrin jararin (Fragment) (*Bothropoides jararaca*)
**Q92043|VM3AA_CROAT**	149.13	14	7	5	Zinc metalloproteinase-disintegrin-like atrolysin-A (Fragment) (*Crotalus atrox*)
**P0DM90|VM32B_GLOBR**	122.17	5	3	2	Zinc metalloproteinase-disintegrin-like brevilysin H2b (*Gloydius brevicaudus*)
**Q7ZZS9|VM2J_PROJR**	118.04	14	6	2	Zinc metalloproteinase/disintegrin (*Trimeresurus jerdonii*)
**C5H5D4|VM33_BOTAT**	90.17	7	2	2	Zinc metalloproteinase-disintegrin-like batroxstatin-3 (Fragment) (*Bothrops atrox*)
**Q90495|VM3E_ECHCA**	55.28	2	2	2	Zinc metalloproteinase-disintegrin-like ecarin (*Echis carinatus*)
**P0CJ41|VSPAF_TRIAB**	304.68	42	13	8	Alpha-fibrinogenase albofibrase (*Cryptelytrops albolabris*)
**P0DJL2|SLA_CRYPP**	295.1	85	15	3	Snaclec purpureotin subunit alpha (*Cryptelytrops purpureomaculatus*)
**P0DJL3|SLB_CRYPP**	264.92	65	10	2	Snaclec purpureotin subunit beta (*Cryptelytrops purpureomaculatus*)
**P0DM38|SLDA_TRIAB**	257.69	49	9	6	Snaclec alboaggregin-D subunit alpha (*Cryptelytrops albolabris*)
**Q71RR4|SLA_TRIST**	256.32	58	10	8	Snaclec coagulation factor IX/factor X-binding protein subunit A (*Viridovipera stejnegeri*)
**P81115|SLBA_TRIAB**	256.21	61	11	3	Snaclec alboaggregin-B subunit alpha (*Cryptelytrops albolabris*)
**Q9YGP1|LECG_TRIST**	212.86	37	9	3	C-type lectin TsL (*Viridovipera stejnegeri*)
**Q71RQ1|SLAA_TRIST**	156.33	28	5	3	Snaclec stejaggregin-A subunit alpha (*Viridovipera stejnegeri*)
**Q71RQ0|SLAB1_TRIST**	115.42	20	3	3	Snaclec stejaggregin-A subunit beta-1 (*Viridovipera stejnegeri*)
**Q8JIV8|SL_DEIAC**	104.16	20	3	2	Snaclec clone 2100755 (*Deinagkistrodon acutus*)
**Q7T2Q0|SLB_ECHML**	100.26	22	3	2	Snaclec EMS16 subunit beta (*Echis multisquamatus*)
**Q6H3D7|PA2HH_TRIST**	254.23	52	14	8	Basic phospholipase A_2_ homolog CTs-R6 (*Viridovipera stejnegeri*)
**Q2YHJ5|PA2AB_TRIPE**	249.27	64	15	8	Acidic phospholipase A_2_ Tpu-E6b (*Trimeresurus puniceus*)
**Q6H3D6|PA2HD_TRIST**	233.17	59	13	7	Basic phospholipase A_2_ homolog Ts-R6 (*Viridovipera stejnegeri*)
**G3DT18|PA2A_BOTMO**	180.26	25	10	7	Acidic phospholipase A_2_ BmooPLA2 (*Bothrops moojeni*)
**A7LAC7|VSP2_TRIAB**	223.03	33	9	2	Thrombin-like enzyme 2 (*Cryptelytrops albolabris*)
**A7LAC6|VSP1_TRIAB**	200.97	37	8	4	Thrombin-like enzyme 1 (*Cryptelytrops albolabris*)
**Q8AY81|VSPST_TRIST**	172.15	23	6	2	Thrombin-like enzyme stejnobin (*Viridovipera stejnegeri*)
**J3SEZ3|PDE1_CROAD**	221.02	28	19	19	Venom phosphodiesterase 1 (*Crotalus adamanteus*)
**Q90W54|OXLA_GLOBL**	211.82	15	9	3	l-amino-acid oxidase (*Gloydius blomhoffii*)
**Q6WP39|OXLA_TRIST**	206.52	14	10	4	l-amino-acid oxidase (*Viridovipera stejnegeri*)
**Q6TGQ8|OXLA_BOTMO**	179.18	23	9	2	l-amino-acid oxidase (Fragment) (*Bothrops moojeni*)
**Q4F867|OXLA_DABSI**	178.26	13	6	2	l-amino-acid oxidase (Fragments) (*Daboia siamensis*)
**X2JCV5|OXLA_CERCE**	133.13	10	6	2	l-amino acid oxidase (*Cerastes cerastes*)
**P60623|CRVP_TRIST**	204.78	46	11	4	Cysteine-rich venom protein (Fragment) (*Viridovipera stejnegeri*)
**P81995|CRVP_CRYPP**	153.46	52	3	3	Cysteine-rich venom protein tripurin (Fragment) (*Cryptelytrops purpureomaculatus*)
**Q8JI39|CRVP_PROFL**	127.32	24	8	3	Cysteine-rich venom protein triflin (*Probothrops flavoviridis*)
**P0DJF5|VSPPA_TRIAB**	177.15	27	8	4	Venom plasminogen activator GPV-PA (*Cryptelytrops albolabris*)
**O13061|VSPB_TRIGA**	165.79	21	5	3	Snake venom serine protease 2B (*Trimeresurus gramineus*)
**Q90YA8|QPCT_GLOBL**	145.59	11	3	3	Glutaminyl-peptide cyclotransferase (*Gloydius blomhoffii*)
**F8S0Z7|V5NTD_CROAD**	106.69	17	6	6	Snake venom 5′-nucleotidase (*Crotalus adamanteus*)
**P0C7P6|BNP_TRIGA**	105.18	17	4	4	Bradykinin-potentiating and C-type natriuretic peptides (*Trimeresurus gramineus*)
**F8S101|PLB_CROAD**	89.21	8	3	3	Phospholipase B (*Crotalus adamanteus*)

**Table 3:** Enzymatic and nonenzymatic proteins from *T. wagleri* crude venom.

Enzymatic	Non-Enzymatic Protein
Snake venom metalloproteinase (SVMP) *	Snake venom C-type lectin (SNACLEC)
Phospholipase A_2_ (PLA_2_)	Waglerin
l-amino acid oxidase (LAAO)	-
Snake venom serine protease (SVSP)	-
Snake venom 5′-nucleotidase	-
Venom phosphodiesterase (vPDE)	-
Phospholipase B (PLB)	-

* Previously not reported in public protein database and/or conventional enzymatic assays.

**Table 4:** Enzymatic and nonenzymatic proteins from *C. purpureomaculatus* crude venom.

Enzymatic	Non-Enzymatic Protein
Snake venom metalloproteinase (SVMP)	Snake venom C-type lectin (SNACLEC)
Phospholipase A_2_ (PLA_2_)	Cysteine-rich venom protein (CRVP)
l-amino acid oxidase (LAAO)	Bradykinin-potentiating and C-type natriuretic peptide (BCNP) *
Snake venom serine protease (SVSP)	-
Glutaminyl-peptide cyclotransferase (QPCT) *	-
Snake venom 5′-nucleotidase	-
Venom phosphodiesterase (vPDE)	-
Phospholipase B (PLB)	-

* Previously not reported in public protein database and/or conventional enzymatic assays.

**Table 5:** Differences in the composition of venom protein families in Malaysian *T. wagleri* and *C. purpureomaculatus* venoms.

Venom Protein Family	*Tropidolaemus wagleri*	*Cryptelytrops purpureomaculatus*
Snake venom C-type lectin (SNACLEC)	23%	19%
Waglerin	15%	-
Phospholipase A_2_ (PLA_2_)	15%	8%
l-amino acid oxidase (LAAO)	8%	10%
Snake venom 5′-nucleotidase	8%	2%
Snake venom serine protease (SVSP)	8%	12%
Venom phosphodiesterase (vPDE)	8%	2%
Phospholipase B (PLB)	8%	2%
Snake venom metalloproteinase (SVMP)	7%	35%
Cysteine-rich venom protein (CRVP)	-	6%
Bradykinin-potentiating and C-type natriuretic peptides (BCNP)	-	2%
Glutaminyl-peptide cyclotransferase (QPCT)	-	2%

## Supplementary Materials

The following are available online at www.mdpi.com/2072-6651/8/10/299/s1, Supplementary Files 1 and 2.
